# 3D bioprinting dual-factor releasing and gradient-structured constructs ready to implant for anisotropic cartilage regeneration

**DOI:** 10.1126/sciadv.aay1422

**Published:** 2020-09-09

**Authors:** Ye Sun, Yongqing You, Wenbo Jiang, Bo Wang, Qiang Wu, Kerong Dai

**Affiliations:** 1 Department of Orthopaedics, The First Affiliated Hospital of Nanjing Medical University, Jiangsu 210029, China.; 2 Clinical and Translational Research Center for 3D Printing Technology, Shanghai Key Laboratory of Orthopaedic Implants, Department of Orthopaedic Surgery, Shanghai Ninth People’s Hospital, Shanghai Jiao Tong University School of Medicine, Shanghai 200011, China.; 3 Department of Nephrology, Affiliated Hospital of Nanjing Medical University, North District of Suzhou Municipal Hospital, Suzhou, China.; 4 State Key Laboratory for Modification of Chemical Fibers and Polymer Materials, College of Chemistry, Chemical Engineering and Biotechnology, Donghua University, Shanghai 201620, China.

## Abstract

Cartilage injury is extremely common and leads to joint dysfunction. Existing joint prostheses do not remodel with host joint tissue. However, developing large-scale biomimetic anisotropic constructs mimicking native cartilage with structural integrity is challenging. In the present study, we describe anisotropic cartilage regeneration by three-dimensional (3D) bioprinting dual-factor releasing and gradient-structured constructs. Dual-factor releasing mesenchymal stem cell (MSC)–laden hydrogels were used for anisotropic chondrogenic differentiation. Together with physically gradient synthetic biodegradable polymers that impart mechanical strength, the 3D bioprinted anisotropic cartilage constructs demonstrated whole-layer integrity, lubrication of superficial layers, and nutrient supply in deep layers. Evaluation of the cartilage tissue in vitro and in vivo showed tissue maturation and organization that may be sufficient for translation to patients. In conclusion, one-step 3D bioprinted dual-factor releasing and gradient-structured constructs were generated for anisotropic cartilage regeneration, integrating the feasibility of MSC- and 3D bioprinting–based therapy for injured or degenerative joints.

## Teaser

3D bioprinting helps to reconstruct your broken knee.

## INTRODUCTION

Articular cartilage is an elastic connective tissue in the joint (*1*). Cartilage injury is extremely common, yet cartilage has limited self-healing capacity because of its low cellularity and avascular nature. Because damage to cartilage leads to knee joint dysfunction, resulting in substantial pain and disability in the arthritic joint, cartilage or joint reconstruction remains a considerable challenge.(*1*). Arthritic joints in clinical practice are replaced by total joint arthroplasty using metallic and synthetic prosthesis (*2*, *3*). Existing joint prostheses do not remodel with host joint tissue and can lead to long-term failure by aseptic loosening or infection (*4*), which could only be addressed by biological regeneration of the joint. Recently, using mesenchymal stem cell (MSC) transplants and then stimulating the directional differentiation into chondrocytes is becoming the method of choice for cartilage repair (*5*, *6*). Clinical studies have shown that joint cartilage damage always extends deeply into the subchondral bone and, thus, causes osteochondral defects in the knee joint, which can alter the joint’s biomechanical properties and influence the long-term performance of the cartilage tissue (*7*), indicating the significance of simultaneous repair of whole-layer anisotropic articular cartilage in successful knee repair. As articular cartilage transitions from the superficial zone to the deep zone, the extracellular matrix (ECM) of the cartilage is characterized by increased oxygen tension and nutrient availability, lower amounts of ECM constituents such as glycosaminoglycans (GAG), and increased presence of a different phenotype of chondrocyte population with hypertrophic and ossification markers such as RUNX2 (Runt-related transcription factor 2) and type X collagen (*8*, *9*). The gradient and anisotropic structure in ECM deposition and cell type provides excellent permeability in deep zone (vessel ingrowth) as well as desired mechanical support (*10*). However, developing biomimetic constructs mimicking the gradient anisotropic structure and the signaling approaches in different layers to induce zonal-dependent chondrogenic differentiation and ECM deposition is very challenging in cartilage repair. Previous studies showed that scaffolds with small pore size (100 to 200 μm) could better promote chondrogenesis in osteochondral regeneration (*11*). However, osteogenesis and angiogenesis were inhibited in these scaffolds with small pore sizes, showing less nutrient diffusion and worse tissue integration by decreased microvessel ingrowth in these scaffolds (*12*). Hydrogel has been reported for cartilage regeneration in many studies (*13*, *14*), yet it is still difficult to construct large-scale tissue structures with hydrogel owing to inadequate structural integrity, mechanical stability, and printability (*12*). Here, we report developing three-dimensional (3D) bioprinted dual-factor releasing and gradient-structured MSC-laden constructs ready to implant for whole-layer cartilage regeneration.

## RESULTS AND DISCUSSION

### 3D bioprinting to fabricate dual-factor releasing and gradient-structured cartilage constructs

**Fig. 1:**
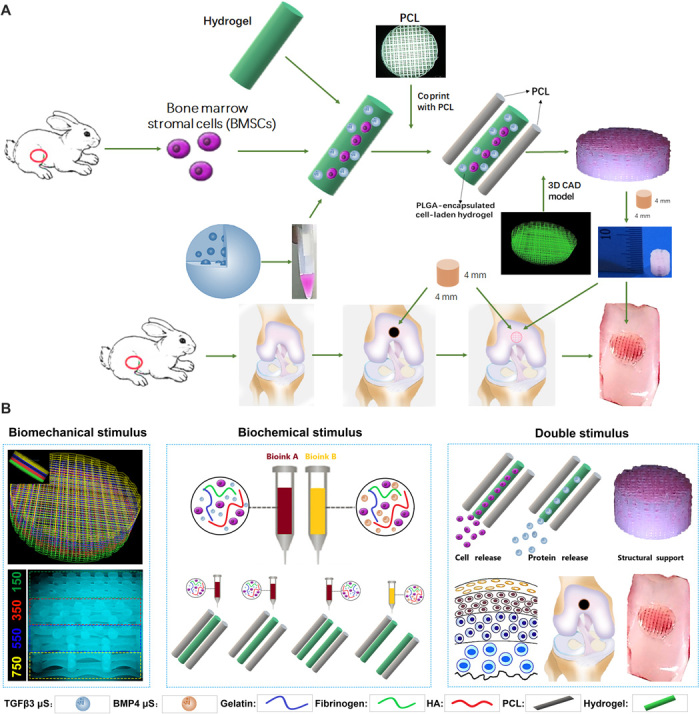
Schematic presentation of the study design and scaffold construction. (**A**) Schematic Illustration of the study design with 3D bioprinted dual-factor releasing and gradient-structured MSC-laden constructs for articular cartilage regeneration in rabbits. Schematic diagram of construction of the anisotropic cartilage scaffold and study design. (**B**) A computer-aided design (CAD) model was used to design the four-layer gradient PCL scaffolding structure to offer BMS for anisotropic chondrogenic differentiation and nutrient supply in deep layers (left). Gradient anisotropic cartilage scaffold was constructed by one-step 3D bioprinting gradient polymeric scaffolding structure and dual protein-releasing composite hydrogels with bioinks encapsulating BMSCs with BMP4 or TGFβ3 μS as BCS for chondrogenesis (middle). The anisotropic cartilage construct provides structural support and sustained release of BMSCs and differentiative proteins for biomimetic regeneration of the anisotropic articular cartilage when transplanted in the animal model (right). Different components in the diagram are depicted at the bottom. HA, hyaluronic acid.

**Fig. 2:**
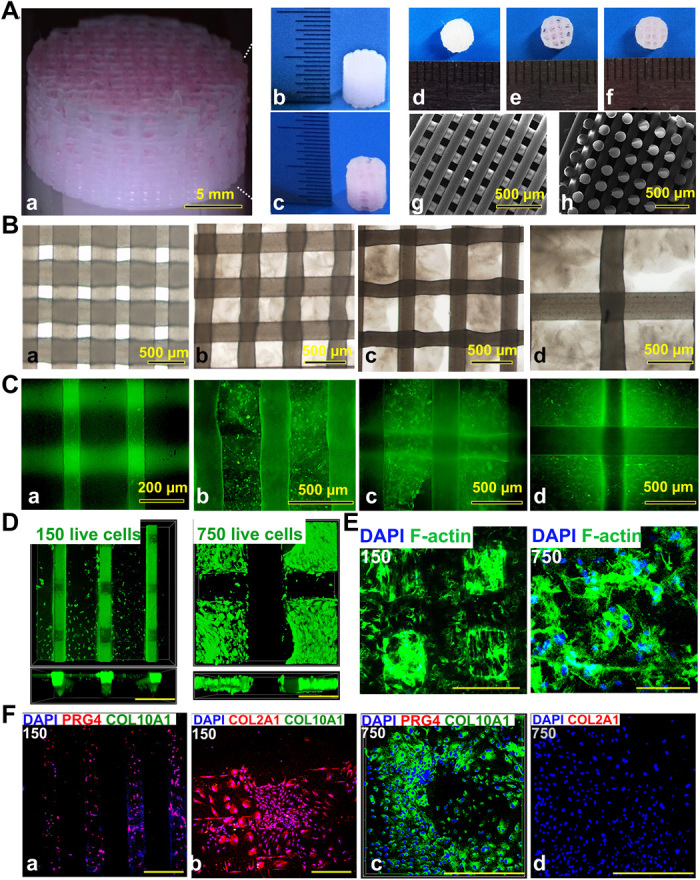
3D bioprinted gradient cartilage scaffold for implantation. (**A**) Gross appearance of (a) human-scale and (b and c) rabbit-scale cartilage scaffold (b, NG with 150-μm spacing; c, NG with 750-μm spacing). Top view of the rabbit cartilage scaffold is also shown (d, NG with 150-μm spacing; e, NG with 750-μm spacing; f, gradient scaffold with 150- to 750-μm spacing) atop of the SEM images (g, horizontal section; h, vertical section) taken for the 150-μm NG scaffold to demonstrate the precise alignment of the PCL fibers in the printed scaffold. (**B**) Deconstruction of the gradient scaffold. The structure of the gradient scaffold was deconstructed into four layers. Microscopic appearance of the hydrogel-PCL composite structure in each layer demonstrated good interconnectivity and delicate, orderly aligned structure for each layer. (**C** and **D**) Good cell viability is shown respectively for superficial and deep layers after printing with live/dead assay (green, live cells; red, dead cells) (C) under a microscope and (D) under a confocal microscope. DAPI, 4′,6-diamidino-2-phenylindole. (**E**) Cell spreading in superficial and deep layers with cytoskeleton staining. (**F**) Immunostaining for cartilage markers in superficial and deep layers. Expression of COL2A1 and PRG4, the lubrication markers, was significantly higher in the superficial layers with small pore size (a and b), while the chondrogenic cells in the deep layers (c and d) mostly presented with hypertrophic phenotype (COL10A1 expression). Photo credit: Ye Sun, First Affiliated Hospital of Nanjing Medical University.

Different joint tissue constructs for joint reconstruction were fabricated using 3D bioprinting as previously reported with organ printing united system (OPUS; Novaprint) (*15*). To better mimic the native cartilage, we incorporated biochemical stimulus (BCS) with different growth factor releasing, and biomechanical stimulus (BMS) with small pore sizes to induce better chondrogenesis to create the dual-factor releasing and gradient-structured cartilage construct in the double stimulus (DS) group. We chose to test the combination of bone morphogenetic protein 4 (BMP4) and transforming growth factor–β3 (TGFβ3) in the cartilage construct in an established knee cartilage defect model given its potential generalizability in the regeneration of complex, inhomogeneous joint tissues. Poly(lactic-co-glycolic acid) (PLGA) (50:50 PLA/PGA) microspheres (μS) were used to deliver TGFβ3 and BMP4 in hydrogel (Fig. 1, A and B). Briefly, poly(ε-caprolactone) (PCL) was molten to fabricate the physically gradient supporting structure for the scaffold, while MSC-laden hydrogel encapsulating PLGA microparticles carrying TGFβ3 or BMP4 in different layers was bioprinted into the microchannels between PCL fibers from different syringes (fig. S1). During plotting, the needle diameter, layer thickness, and speed for PCL printing were kept constant at 200 μm, 200 μm, and 180 mm/min, respectively. The fiber spacing was kept constant at 150 μm (BMS group) or 750 μm (BCS group) for nongradient (NG) scaffolds and varied gradually from 150 to 750 μm throughout the gradient scaffolds (DS group) (fig. S1). The gradient microchannels between PCL range gradually from 150 μm wide from the superficial zone of the cartilage, providing enough mechanical properties and smaller compartments favoring articular chondrocyte differentiation (*11*, *16*), to 750 μm wide in the deepest zone of the cartilage construct, maximizing diffusion of nutrients with better microvessel ingrowth and offering higher oxygen stress in the deep zone (Fig. 1B) (*12*). The fiber spacing was changed by 200 μm every millimeter. The scaffolds were plotted in blocks of 4 × 4 × 4 mm for rabbit cartilage construct and 14 × 14 × 14 mm for human cartilage construct (Fig. 2A and movie S1).

Recombinant human TGFβ3 (rhTGFβ3) and rhBMP4 were microencapsulated in PLGA μS (fig. S1) (*17*). TGFβ3 and BMP4 μS were mixed in the cell-laden hydrogel (table S1), respectively, and printed into the microchannels between PCL fibers with different syringes (Fig. 2B and fig. S1). To chemically simulate the hypertrophic layer in native cartilage, we used PLGA^BMP4^-encapsulated MSC-laden hydrogel in the deepest layer with a 750-μm PCL fiber spacing, while PLGA^TGFβ3^ was used for the other three layers of the cartilage construct. Scanning electron microscopy (SEM) images of PLGA μS were taken, showing a less than 2-μm diameter for most of the PLGA μS. The PLGA-encapsulated MSC-laden hydrogel also showed nice printability as demonstrated (Fig. 2B and fig. S1A).

The final product of the human and rabbit cartilage construct demonstrated good interconnectivity and delicate, orderly aligned structure under the microscope, SEM, and in gross appearance for both PCL fibers and the printed hydrogel in between (Fig. 2, B to D). To validate μS distribution in MSC-laden hydrogel, fluorophore-conjugated rhodamine was encapsulated into PLGA μS and delivered to the hydrogel. At day 7, PLGA^rhodamine^ μS showed well-proportioned distribution and minimal cell toxicity in the hydrogel printed between the PCL fibers under a confocal microscope (Fig. 2, C and D, and fig. S1B). Immunostaining for cartilage markers in the gradient scaffold was performed (Fig. 2, E and F). Resembling the native cartilage, the expression of COL2A1 (Collagen Type II Alpha 1 Chain) and PRG4 (Proteoglycan 4), the lubrication marker, was significantly higher in the superficial layers with small pore size, while the chondrogenic cells in the deep layers mostly presented with hypertrophic phenotype (COL10A1 expression) (Fig. 2F and fig. S2). Moreover, the compressive Young’s modulus of the NG-150 scaffold and the gradient scaffold were similar to that of the native cartilage and significantly higher than that of the NG-750 scaffold (fig. S3), demonstrating that smaller PCL fiber spacing plays an important role in enhancing the mechanical properties of the PCL-hydrogel composite scaffolds. In biomimetic regeneration of native articular cartilage, the gradient scaffold could provide anisotropic chondrogenesis in different layers and structural support for the newly formed cartilage tissue in compression, and allow nutrient supply and vessel ingrowth in the deep layers.

### Effects of dual-factor releasing nanoparticles on the viability and proliferation of BMSCs in the scaffolds in vitro

**Fig. 3:**
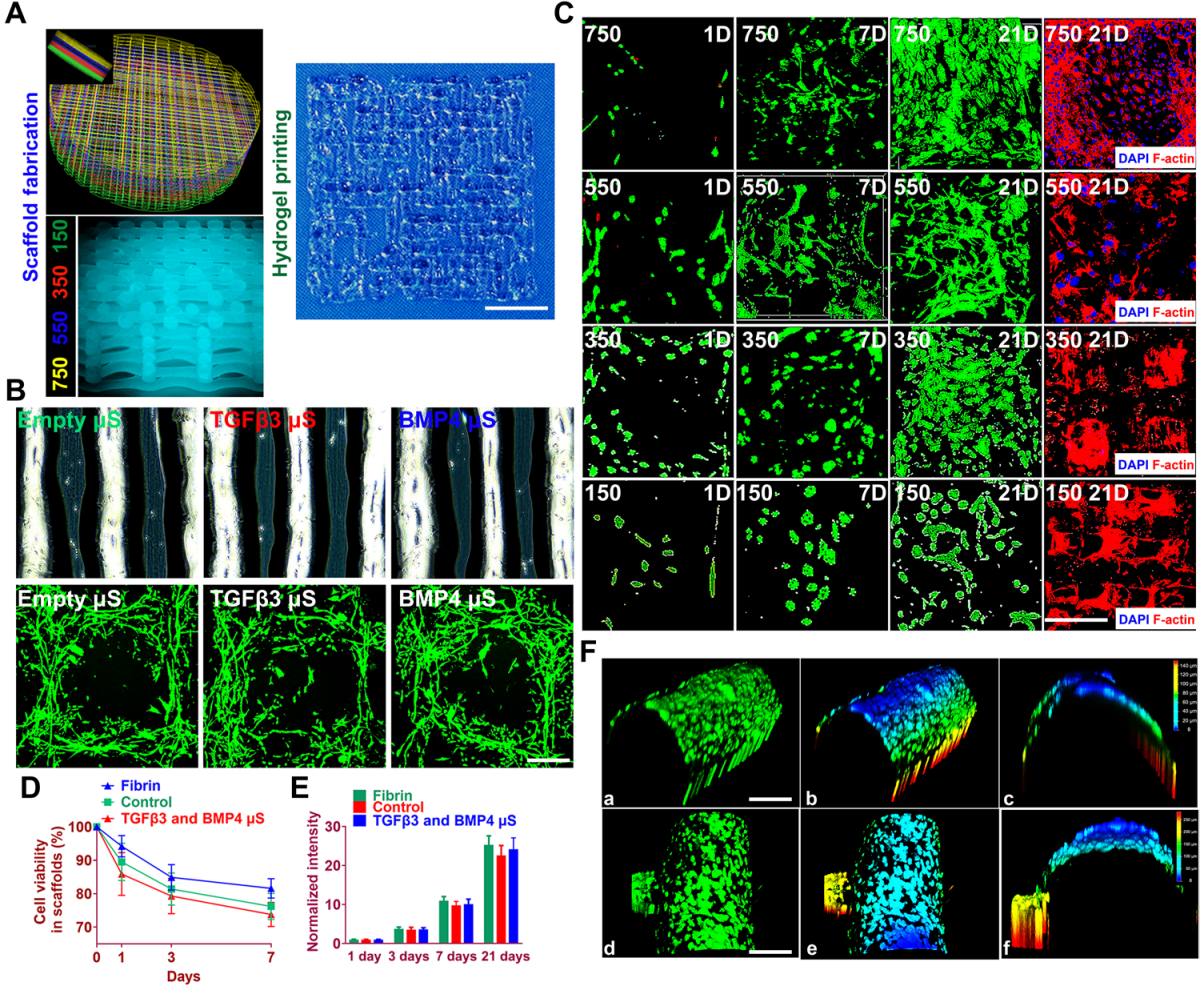
Cell viability and anchoring in the printed anisotropic scaffold. (**A**) Schematic of anisotropic cartilage scaffold construction with fabrication of gradient scaffolding structure (left) and large-scale printing of aligned protein-releasing BMSC-laden hydrogel (right). Scale bar, 1 mm. (**B**) Gross appearance of PLGA μS–encapsulated BMSC-laden hydrogel under a microscope (top). Printed cell-laden hydrogel causes cell alignment in a longitudinal direction of the printed paths, forming a reticular network with cell interaction (bottom). (**C**) Live/dead cell assays showed ≥95% cell viability maintained through day 1 to 21 for all four layers with gradient spacing (4th row, 150-μm spacing; 3rd row, 350-μm spacing; 2nd row, 550-μm spacing; 1st row, 750-μm spacing). Immunostaining of cytoskeleton (rightmost column) showed cell spreading both in the hydrogel and on the PCL fibers throughout the four layers of the construct. Scale bar, 500 μm. (**D** and **E**) Quantified cell viability and proliferation in the printed scaffolds. (**F**) Cell anchoring in the scaffolds. (a to c) At day 21, good 3D anchoring to the PCL fiber cylinder was observed for the MSC cells released from the hydrogel. (d to f) Similar cell anchoring was observed for PCL fibers in adjacent layers. (b), (c), (e), and (f) are 3D demonstration of cell anchoring in (a) and (d), respectively. Scale bars, 100 μm. Photo credit: Ye Sun, First Affiliated Hospital of Nanjing Medical University.

To examine the effects of BMP4, TGFβ3, and their μS on bone marrow stromal cell (BMSC) viability and proliferation, we cultured BMSCs in the composite hydrogel for 7 days (fig. S4). Spheres showed controlled release of TGFβ3 first, followed by BMP4. Relatively rapid TGFβ3 release in the three layers with smaller PCL fiber spacing and slower release of BMP4 in the deepest layer were sustained over 60 days in vitro (fig. S5). Similar viability and proliferation rate of BMSCs were demonstrated for BMP4 and TGFβ3 compared with control through 7 days in the hydrogel (fig. S4, A, C, and D). Compared with empty μS, μS encapsulating BMP4 and TGFβ3 also showed minimal toxicity to BMSC viability and proliferation in the hydrogel (fig. S4, B, E, and F). Cell viability and proliferation were further examined in the printed scaffolds (Fig. 3, A to E). Scaffold fabrication with gradient structure (Fig. 3A, left) and delicate alignment of hydrogel printing (Fig. 3A, right) were separately conducted. Printed cell-laden hydrogel causes cell alignment in a longitudinal direction of the printed paths, forming a reticular network with cell interaction (Fig. 3B). The PCL pillar structure in the final construct further stabilized the 3D printed BMSC organization, inducing a compaction phenomenon of the patterns of cell alignment in the cell-laden hydrogel (Fig. 3C). Survival of BMSCs throughout the final cartilage construct with gradient structure was examined at 60 min (day 0), 1 day, 7 days, and 21 days after printing (Fig. 3, I to K). Live/dead cell assays showed ≥95% cell viability on day 0, which was maintained over 75% through days 3 to 21 (Fig. 3D). Cell proliferation, assessed using the alamarBlue assay system, increased over a 21-day period, similar to the proliferation of control cells encapsulated in a fibrin construct (Fig. 3E). Immunostaining of cytoskeleton showed cell spreading, both in the hydrogel and the PCL fibers throughout the four layers of the construct (Fig. 3C). At day 21, good 3D anchoring to the PCL fiber cylinder was observed for the BMSCs released from the hydrogel (Fig. 3F). These data indicate that the one-step 3D bioprinted dual-factor releasing and gradient-structurally optimized cartilage scaffold preserved cell viability during the printing process and provided a favorable microenvironment for BMSC proliferation, spreading, and condensation for differentiation into chondrocytes in vitro.

### Spatiotemporally released rhTGFβ3 and rhBMP4 induced cartilaginous matrix formation in 3D bioprinted gradient-structured scaffold in vitro

**Fig. 4:**
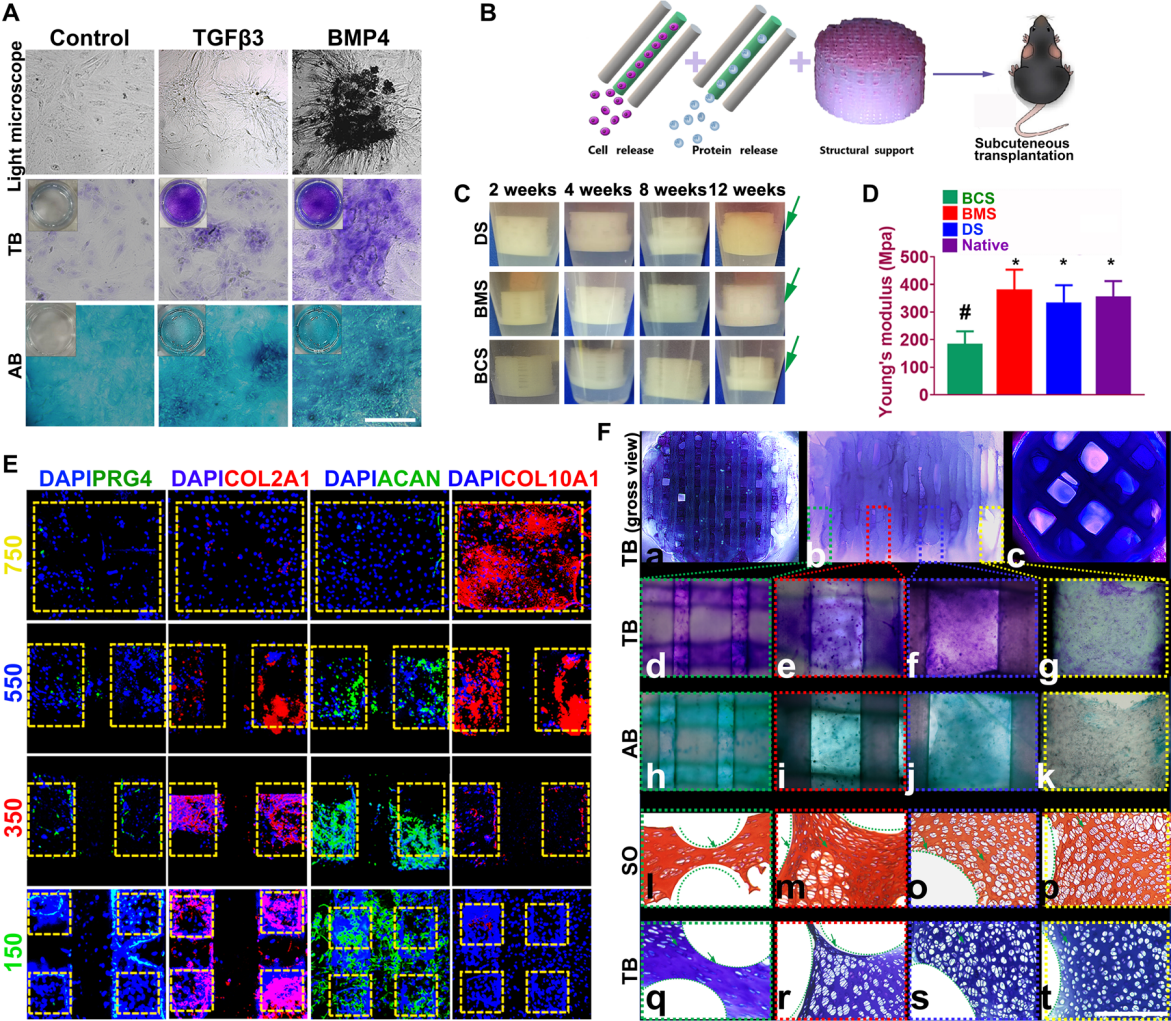
Dual-factor releasing induced cartilaginous matrix formation in 3D bioprinted gradient-structured scaffolds. (**A**) Chondrogenic differentiation of condensed rMSCs with toluidine blue (TB) and alcian blue (AB) staining. (**B**) Scaffolds were transplanted subcutaneously for 12 weeks. (**C**) To validate the cartilage-generating capability, scaffolds were incubated and observed for 12 weeks in vitro, indicating better cartilage-generating potential for the physically gradient protein-releasing scaffold (movie S2). (**D**) Young’s modulus of the scaffolds compared with native cartilage after 12 weeks. Data are presented as averages ± SD (*n* = 6). *P < 0.05 between the NG-750 group and other groups; ^#^*P* < 0.05 between the native cartilage group and other groups. (**E**) In the generated cartilage tissues, spatiotemporally released dual-factors induced zone-specific expression of PRG4, aggrecan, and collagens II and X and showed resemblance with native joint cartilage. (**F**) (a to c) Toluidine blue staining of the 3D printed cartilage constructs (a, top view; b, side view; c, bottom view) after culture in chondrogenic medium for 6 weeks in vitro. (d to g) Toluidine blue and (h to k) alcian blue staining was applied for each layer of the gradient scaffold. (l to p) Safranin O (SO) and (q to t) toluidine blue staining of cartilage tissue between PCL fibers (green curved line) in different layers of the 3D printed cartilage constructs after subcutaneous implantation. Photo credit: Ye Sun, First Affiliated Hospital of Nanjing Medical University.

Before in vivo application of the scaffold, we ascertained whether spatiotemporal delivery of rhTGFβ3 and rhBMP4 induced layer-specific BMSC differentiation into chondrocytes that present with hyaline articular and hypertrophic phenotype. Articular chondrocytes with hyaline and hypertrophic phenotype were first derived from rabbit BMSCs in vitro. Hyaline chondrocytes concurrently produced both aggrecan and type II collagens, while hypertrophic chondrocytes produced type I collagen and type X collagen. Sequential application of rhTGFβ3 for 2 weeks in culture, followed by rhTGFβ3 for another 4 weeks (TGFβ3 group), induced differentiation of BMSCs into chondrocytes that synthesized aggrecan and type II collagens, suggesting hyaline articular chondrocyte-like cells. BMSCs sequentially treated with rhTGFβ3 and rhBMP4 demonstrated significantly higher type I collagen, type X collagen, and aggrecan protein expressions than the control (Fig. 4A and fig. S6). Moreover, cells in the TGFβ3-induced tissue were fibroblastic, whereas those induced with BMP4 were larger and arranged in a cobblestone pattern (Fig. 4A), similar to hypertrophic chondrocytes previously generated in culture (*5*). Condensation of BMSCs that indicated differentiation was observed at 4 weeks (fig. S6B). Both treatments induced BMSC differentiation and yielded a cartilaginous matrix that stained positively for toluidine blue and alcian blue in condensed BMSCs, indicative of a proteoglycan-rich, cartilage-like ECM.

Cartilage scaffolds incorporating rhTGFβ3 and rhBMP4 for spatiotemporally controlled release were also examined in different groups of scaffolds transplanted in vivo subcutaneously for 12 weeks (Fig. 4, B to F). To validate the cartilage-generating capability of the composite scaffold, the protein-carrying scaffolds were incubated and observed for 12 weeks in vitro (Fig. 4C). All scaffolds, physically gradient or NG, showed cartilage-like tissue development surrounding the scaffolds, whereas the BCS and BMS scaffolds developed 1/4 to 1/3 thickness cartilage tissue, while the DS scaffold showed almost full-thickness coverage of cartilage-like tissue around the construct (movie S2), indicating a significantly better cartilage-generating potential in vitro and a better prospect of its cartilage matrix integration in vivo for the physically gradient protein-releasing scaffold (Fig. 4C). The compressive Young’s modulus of the BMS scaffold and the DS scaffold were similar to that of the native cartilage and significantly higher than that of the BCS scaffold with large pore sizes (Fig. 4D), demonstrating that smaller PCL fiber spacing plays an important role in enhancing the mechanical properties of the PCL-hydrogel composite scaffolds. The enhanced mechanical properties are promising for biomimetic regeneration of native articular cartilage and provide structural support for the newly formed cartilage tissue.

After 12 weeks in vivo, spatiotemporally released rhTGFβ3 and rhBMP4 in the DS scaffold induced zone-specific expression of PRG4, aggrecan, and collagen II and X assayed with immunofluorescence, showing resemblance with native joint cartilage (Fig. 4E). Superficial zone marker PRG4, with a gradient manner throughout the four layers, was presented mainly in the superficial layer with the smallest PCL compartments (Fig. 4E, first column, 150 μm × 150 μm). Abundant cartilaginous matrix with collagen type II and aggrecan was present in a gradient manner primarily in the superficial layers with TGFβ3 delivery, whereas hypertrophic marker collagen type X was primarily expressed in the deepest zone (Fig. 4E, second to fourth columns). Cartilaginous matrix was demonstrated and stained positive for toluidine blue for the scaffold (Fig. 4F, a to c). To determine the production of GAG in each layer of the gradient scaffold, we applied toluidine blue staining (Fig. 4F, d to g) and alcian blue staining (Fig. 4F, h to k). The whole gradient scaffold body stained positive (Fig. 4F, a to c), with a gradient staining intensity from the superficial layer to the deepest layer (Fig. 4F, d to k), indicating a gradient cartilaginous matrix formation resembling the native cartilage matrix. Safranin O staining and toluidine blue staining of the generated cartilage tissue sections showed the production of a cartilaginous matrix between PCL fibers in different layers of the 3D printed cartilage constructs after subcutaneous implantation in vivo (Fig. 4F, j to s). The chondrocytes in the newly formed tissue demonstrated similar morphological characteristics to those in native cartilage. A large fraction of generated chondrocytes in the TGFβ3-induced tissue were fibroblastic, whereas those induced with BMP4 in the deepest layers were larger and arranged in a cobblestone pattern, similar to hypertrophic chondrocytes generated in the culture plate (Fig. 4F, l to t). All cells located within typical chondrocyte lacunae, surrounded by cartilaginous matrix.

### Dual-factor releasing and gradient-structured scaffold demonstrated better repairing effect of cartilage in rabbit knee cartilage defect model in vivo

**Fig. 5:**
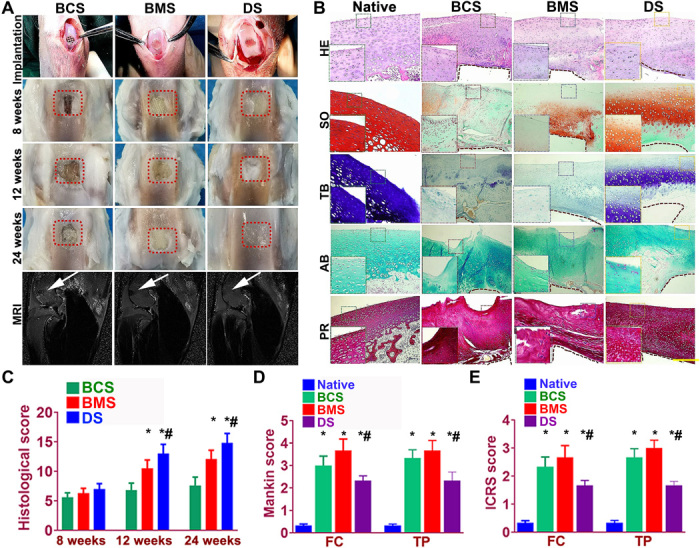
Dual-factor releasing and gradient-structured cartilage scaffold demonstrated better repairing effect of anisotropic cartilage in rabbit knee cartilage defect model in vivo. (**A**) Scaffold implantation process and gross appearance of the repair cartilage at 8, 12, and 24 weeks. MRI was made for the operated knee joint (fifth row), demonstrating significant better resolution of subchondral edema and healing of the articular surface (white arrowheads) for joint transplanted with DS scaffolds. (**B** to **F**) Chondroprotective effects of the scaffolds were compared by (B) histological scoring evaluation of the repaired cartilage tissue during in vivo implantation. (C) Mankin score and (D) ICRS (International Cartilage Repair Society) histological score of articular cartilage in the femoral condyle (FC) and tibial plateau (TP) in both groups with scaffold implantation. *P < 0.05 between the native group and other groups. ^#^*P* < 0.05 between the BCS group and the DS group. Data are presented as averages ± SD (*N* = 6). (A) Histomorphological analysis of the neocartilage tissue at 24 weeks. PR, picrosirius red. The left bottom panels are higher-resolution pictures of the formed neocartilage outline in the colored square boxes. (a to e) Sections were stained with (a) H&E, (b) Safranin O, (c) TB, and (d) AB staining to indicate the presence of proteoglycans in different groups compared with native cartilage. (e) Picrosirius red was used to stain collagens I and III. The brown irregular area at the interface under the formed neocartilage was undegraded PCL material as supporting structure for the scaffolds. Photo credit: Ye Sun, First Affiliated Hospital of Nanjing Medical University.

Rabbits were used as animal models to evaluate the knee repair capacity of the cartilage scaffolds. Cartilage scaffolds were constructed by one-step 3D bioprinting gradient polymeric supporting structure and different protein-releasing composite hydrogels with bioinks encapsulating BMSCs with BMP4 or TGFβ3, providing structural support and sustained release of BMSCs and differentiative proteins for biomimetic regeneration of the native articular cartilage (Fig. 5). As shown in Fig. 5A (first row), a full-thickness cartilage defect was created in the knee joint. The scaffold was implanted into the defect to test for cartilage tissue regeneration. Cartilage repair with the DS scaffold showed much better gross appearance at 8, 12, and 24 weeks compared with the BCS and BMS scaffolds (Fig. 5A, second to fourth rows). During the 24-week posttransplantation period, magnetic resonance imaging (MRI) was made for the operated knee joint, demonstrating significantly better resolution of subchondral edema and healing of the articular surface after 24 weeks for the DS group (Fig. 5A, fifth row). In addition, the chondroprotective effects of the scaffolds were compared (*18*). The gradient scaffold group showed better chondroprotective effects with a significantly higher histological grading compared with the NG groups over the 24 weeks in vivo (Fig. 5, B to E). Better repairing effects were demonstrated with gradient scaffolds compared with NG groups over 24 weeks (Fig. 5, B to E). Compared with the control group, the gradient group also showed better cartilage regeneration capabilities (fig. S7) and chondroprotection with significantly minor damage to the femoral condyle and tibial plateau (Fig. 5, D and E). Examination of intra-articular inflammatory response showed no significant difference in interleukin-1 and tumor necrosis factor–α level among different groups, maintaining at a relatively low level during the 24-week cartilage healing (fig. S8, A and B, and table S2). After the 24-week healing, histomorphological analysis was conducted for the generated cartilage. As shown in Fig. 5B, the DS scaffold regenerated fully hyaline-like cartilage in the defect site as evidenced by intense staining for GAGs and better cell filling in hematoxylin and eosin (H&E) staining (Fig. 5B). Type 1 and III collagens were also demonstrated in the regenerated cartilage with picrosirius red staining and compared with the native cartilage (Fig. 5B). Immunohistochemical staining of markers (PRG4 and type II and X collagens) for chondrocyte phenotype was conducted in the generated cartilage tissue sections in different groups compared with the native cartilage (fig. S8C). In the superficial zone, only the DS scaffolds showed PRG4 staining in the superficial chondrocytes in the generated cartilage tissue. Meanwhile, gradient expression of type II and X collagens, resembling the native cartilage, was also demonstrated from the superficial zone to the deep zone of the newly formed cartilage in the DS group, indicating successful construction of the anisotropic layered cartilage with different chondrocyte phenotypes and gradient ECM deposition by the 3D bioprinted dual-factor releasing and gradient-structured MSC-laden scaffold. Furthermore, neocartilage in the DS group showed more similar appearance to normal cartilage than other groups (Fig. 5B and fig. S8C). The above results indicated that the DS anisotropic scaffold had a better cartilage-repairing effect than the BCS or BMS groups and maintained better joint function after transplantation.

### Dual-factor releasing and gradient-structured scaffold restored the anisotropic properties of native cartilage and better microvessel ingrowth

**Fig. 6:**
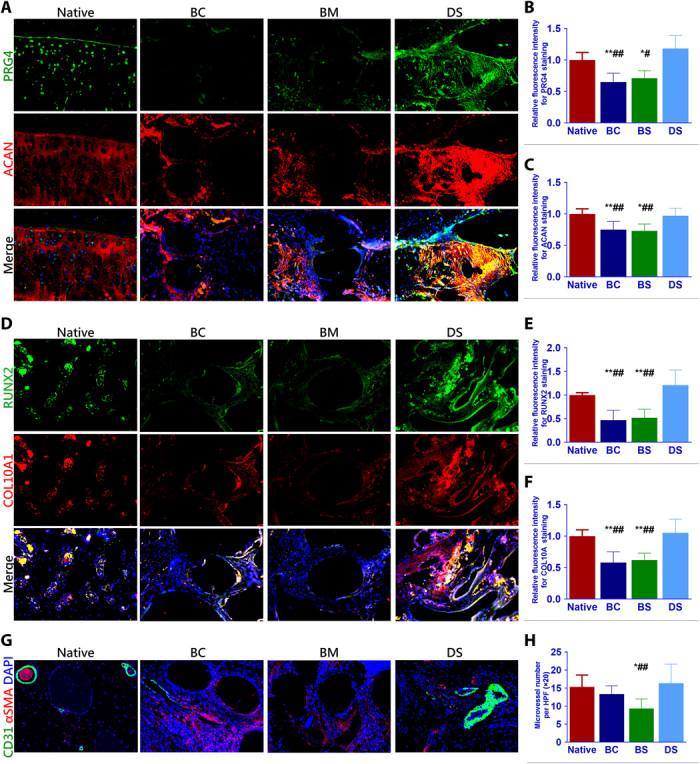
Dual-factor releasing and gradient-structured scaffold restored the anisotropic properties of native cartilage and better microvessel ingrowth. (**A** to **C**) In the superficial layer, immunostaining demonstrated greater PRG4 and ACAN expression in the DS group and the native cartilage compared with other two groups. (**D** to **F**) Meanwhile, higher expression of ossification markers (RUNX2 and COL10A1) were also observed for the group with implanted dual-factor releasing and gradient-structured scaffold in deep layers. (**G** and **H**) Moreover, the DS scaffold could better promote microvessel ingrowth compared with the group with small pore sizes, indicating better nutrient supply and tissue integration with large pore sizes in the deep zone. *P < 0.05 between the native group and other groups. ^#^*P* < 0.05 between the DS group and other groups. BC, biochemical stimulus; BS, biomechanical stimulus. **P < 0.01; ^##^*P* < 0.01.

As native articular cartilage transitions from the superficial zone to the deep zone, different phenotypes of chondrocyte population were presented with higher lubrication and GAGs (PRG4, ACAN expression) in the superficial layers and ossification (RUNX2, COL10A1 expression) in the deep layers. In the present study, we further tested the anisotropic properties of the generated cartilage and compared it with the native cartilage. In the superficial layer, immunostaining demonstrated greater PRG4 and ACAN expression in the DS group and the native cartilage compared with other two groups (Fig. 6, A to C). Meanwhile, higher expression of ossification markers (RUNX2 and COL10A1) were also observed for the group with implanted dual-factor releasing and gradient-structured scaffold (Fig. 6, D to F). These results indicate that the dual-factor releasing and gradient-structured scaffold could better restore the anisotropic properties of the native cartilage with different chondrogenic and ossification markers in specific layers. Moreover, resembling the ingrown microvessels in the deep layers of the native cartilage, the DS scaffold could better promote microvessel ingrowth compared with the group with small pore sizes, indicating better nutrient supply and tissue integration with large pore sizes in the deep zone (Fig. 6, G and H).

In conclusion, we have generated 3D bioprinted anisotropic constructs with structural integrity for joint reconstruction and articular cartilage regeneration and further tested the functional knee articular cartilage construct in a rabbit cartilage defect model with 6-month follow-up. Human-scale cartilage constructs with the structural integrity needed and that are ready for surgical implantation were created by sequentially printing protein-releasing and MSC-laden hydrogels with synthetic PCL polymer with gradient structures, a technique that could also be applied to the regeneration of the whole joint. In previous studies, relative nonuniformity was possible when hydrogel was printed alone without PCL as scaffolding support. Although hydrogel could serve as a carrier of cells and growth factors, it alone was quite not suitable for construction of complex biomimetic tissues with required mechanical properties. The combined printing with PCL scaffolding offered the uniformity for the hydrogel and the mechanical properties needed for in vivo study. In the present study, the cell-laden hydrogel allows well-proportioned distribution of MSCs and the protein-encapsulated μS and thus protects cell viability and promotes its differentiation and expansion in the scaffold (*17*). Meanwhile, the adjacent PCL scaffolding provides adequate mechanical support and architectural integrity, offering a stable microenvironment for the 3D anchored MSC cells within the hydrogel to differentiate and form the tissue with their secreted cartilage matrix that replaces the hydrogel as it slowly degrades (*15*).

However, the release of the growth factors from the embedded μS was not tracked in vivo after the scaffold transplantation. The intra-articular environment in vivo would definitely lead to faster disintegration of the μS in the hydrogel. In this case, the PCL scaffolding would offer a much more stable microenvironment for cell and growth factor release than hydrogel alone. Lineage tracing studies have provided compelling evidence that articular chondrocytes derive from interzone cells in regions of condensing chondrogenic mesenchyme (*19*), similar to our observations that the MSCs, in the presence of TGFβ3 and BMP4, condense in the small compartments with surrounding PCL fibers as supporting structure and develop into articular chondrocytes that express genes expressed in cartilage layers. The MSC-derived articular chondrocytes were able to generate and maintain stable cartilage phenotype in vivo when transplanted into the knee defect site. The ECM composition of TGFβ3- or BMP4-induced cartilage tissues in the bioprinted scaffold shared many characteristics of native articular cartilage, including the gradient expression of type II collagen, superficial localization of PRG4, and abundant presence of type X collagen in the deep zone, indicative of regenerated superficial zone articular cartilage and deep zone hypertrophic cartilage in the constructs. In summary, we have generated 3D bioprinted constructs with structural integrity for joint reconstruction and articular cartilage regeneration and further tested the functional knee articular cartilage construct in a rabbit cartilage defect model with 6-month follow-up. Generating 3D bioprinted functional constructs as prosthesis for joint replacement or cartilage repair provides an opportunity to integrate the feasibility of MSC- and 3D bioprinting–based therapy for injured or degenerative joints. Evaluation will be needed to assess the function of the joint constructs in animal experiments and whether the functional cartilage phenotypes could be sustained in daily function. For translation, we envision the surgeons could incorporate surgery and 3D bioprinting by performing a mini-invasive arthroscopy procedure to replace the damaged or degenerated articular cartilage with 3D bioprinted cartilage scaffold or by performing joint replacement surgery using 3D bioprinted joint scaffolds.

## MATERIALS AND METHODS

### In vitro experiments

BMSCs were isolated from rabbit bone marrow aspirates. Briefly, marrow aspirates (20-ml volume) were harvested and immediately transferred into plastic tubes. Isolated rMSCs were expanded in α–minimum essential medium containing fetal bovine serum (10%), d-glucose (4.5 mg/ml), nonessential amino acids (0.1 mM), sodium pyruvate (1 mM), Hepes buffer (100 mM), penicillin (100 Ul/ml), streptomycin (100 μg/ml), and l-glutamate (0.29 mg/ml). Medium was changed twice a week, and rMSCs were used at passage 2 for the following experiments. TGFβ3 (10 ng/ml) was added in the medium for 2 weeks, and then TGFβ3 was replaced with BMP4 (50 ng/ml) in some of the cultures for another 4 weeks. Medium was also changed twice a week. Immunofluorescence staining of chondrogenic markers (Col1A1, Col2A1, Aggrecan, and Col10A1) was conducted to compare the generated chondrocyte phenotype and observed under confocal microscopy (Leica, Japan). The expression of chondrogenesis markers (SOX9, Col1A1, and Col2A1), superficial zone chondrocyte markers (ACAN, PRG4, CILP2, GDF5, and Col22A1), and deep zone chondrocyte markers(Col10A1, RUNX2, and ALP) after TGFβ3 or BMP4 incubation for 6 weeks was analyzed by real-time polymerase chain reaction (RT-PCR) using an ABI 7300 RT-PCR system (Applied Biosystems, USA). Six-week-old tissues generated under both conditions were stained with toluidine blue and alcian blue for proteoglycan production. The stained images were taken using a light microscope (Leica Microsystems, Germany).

### Fabrication of scaffolds

Different joint tissue constructs for joint reconstruction were fabricated using 3D bioprinting with OPUS (Novaprint). 3D bioprinting cell-laden hydrogels together with biodegradable polymers was conducted for specific articular joint. The motion program and alignment of cell-laden hydrogel and PCL fibers were demonstrated in the printing process of anisotropic cartilage tissues in movie S1. Bioprinting rabbit-derived MSC-laden hydrogels together with physically and chemically gradient biodegradable polymers was conducted for knee cartilage repair using OPUS. The rMSCs suspension (a total of 1 × 10^7^ cells) was loaded into the composite hydrogel (table S1). The printing chamber was kept at a constant 17°C. The native cartilage structure inspired us to produce four-layer 3D structures by placing together cell-laden hydrogel and PCL (~100-μm diameter for hydrogel and ~200-μm diameter for PCL) to construct a composite cartilage scaffold (*17*). Needle sizes for the hydrogel and PCL were 100 and 200 μm, respectively. Briefly, PCL was molten (~60°C) to fabricate the physically gradient supporting structure for the scaffold, while MSC-laden hydrogel (~37°C) encapsulating PLGA microparticles carrying TGFβ3 or BMP4 in different layers was bioprinted into the microchannels between PCL fibers from different syringes (movie S1). During plotting, the needle diameter, layer thickness, and speed for PCL printing were kept constant at 200 μm, 200 μm, and 180 mm/min, respectively, as previously reported (*15*). The extrusion pressure for PCL and hydrogel was 1.2 to 1.8 kPa and 0.5 to 0.8 kPa, respectively. The fiber spacing was kept constant at 150 or 750 μm for NG scaffolds and varied gradually from 150 to 750 μm throughout the gradient scaffolds. The gradient microchannels between PCL range gradually from 150 μm wide from the superficial zone of the cartilage to 750 μm wide in the deep zone of the cartilage construct. The fiber spacing was changed every millimeter. The scaffolds were plotted in blocks of 4 × 4 × 4 mm for rabbit cartilage construct and 14 × 14 × 14 mm for human cartilage construct.

rhTGFβ3 and rhBMP4 were microencapsulated in PLGA (50:50 PLA/PGA) μS to deliver TGFβ3 (20 ng/ml) and BMP4 (100 ng/ml) in hydrogel as previously described (*15*, *17*). TGFβ3 and BMP4 μS were mixed in the cell-laden hydrogel (table S1), respectively, and printed into the microchannels between PCL fibers with different syringes. To chemically simulate the deep layer in native cartilage, PLGA^BMP4^-encapsulated MSC-laden hydrogel was used in the deepest layer with a 750-μm PCL fiber spacing, while PLGA^TGFβ3^ was used for the other three layers of the cartilage construct. Generated PLGA μS was shown with SEM. Printability was also shown with a test run for the PLGA-encapsulated MSC-laden hydrogel. Release kinetics of TGFβ3 and BMP4 from PLGA μS were measured by incubating μS (10 mg/ml) encapsulating TGFβ3 (0.1% bovine serum albumin) or BMP4 [in phosphate-buffered saline (PBS)] at 37°C with mild agitation for up to 60 days. Upon centrifugation at 2500 revolutions per minute for 5 min, supernatant of the PLGA μS incubation solution was collected. Released TGFβ3 and BMP4 concentration was measured using enzyme-linked immunosorbent assay kits following the manufacturer’s protocols (*15*). To validate μS distribution in MSC-laden hydrogel, fluorophore-conjugated rhodamine was encapsulated into PLGA μS and delivered to the hydrogel. At day 7, PLGA rhodamine μS and cell viability (live/dead assay) in the hydrogel was observed under a confocal microscope.

To validate the cartilage-generating capability of the composite scaffold, the protein-carrying scaffolds were incubated and observed for 12 weeks in vitro. Photographs of cartilage-like tissue development surrounding the scaffolds were taken to show the cartilage-generating potential in vitro of the scaffolds. Mechanical measurements on scaffolds and native cartilage were carried out with a single-column static instrument (Instron 5843, USA) equipped with two flat compression stages and a 10-N load cell.

### Partition analysis

To see the differences within the rMSCs cultured in the different areas of the gradient scaffolds, after 6 weeks under differentiation conditions, the constructs were collected, washed three times with PBS, and cut in four portions of 1 mm in height. The images of each layer were taken using a light microscope. The viability of the BMSCs on the scaffolds were analyzed with live/dead assay and observed under confocal microscopy for 3, 7, and 21 days, while the morphology of cells was observed under confocal microscopy at end point (21 days). Briefly, The MSCs in the scaffold were fixed with 4% paraformaldehyde and treated with rhodamine phalloidin (Thermo Fisher Scientific, USA) to stain the F-actin for 1 hour and incubated with DAPI (Thermo Fisher Scientific, USA) to stain the nucleus for 5 min in turn. Cell proliferation in the constructs was assessed with alamarBlue assay kit (DAL1100; Life Technologies) according to the manufacturer’s instruction as previously described (*12*).

Biochemical studies were performed to the full and partitioned scaffolds. Toluidine blue and alcian blue staining were applied to determine the production of GAGs in each layer of the gradient scaffold. The sections for the different layers were prepared and then treated with Safranin O and toluidine blue staining to identify GAG formation in each layer. Immunofluorescence staining of chondrocyte markers (PRG4, Col2A1, aggrecan, and Col10A1) was conducted for layer-specific chondrogenesis and observed under confocal microscopy.

### Animal experiment

Different groups of scaffolds were transplanted under the dorsal skin of nude mice in vivo subcutaneously for 12 weeks. The cartilage scaffolds were retrieved after 12 weeks in vivo, and zone-specific expressions of PRG4, aggrecan, and type II and X collagens were assayed with immunofluorescence. GAG production was determined with toluidine blue and alcian blue staining.

Adult male New Zealand white rabbits weighing 3.0 to 3.5 kg were used for the study in vivo. Rabbits were randomized into three groups (two knees of each rabbit were used): NG-750 (BCS group), NG-150 scaffold (BMS group), and the gradient scaffold (DS group). After anesthesia, the knee joint of the rabbits was exposed after dislocating the patella. A cylindrical defect (4-mm diameter, 4-mm depth) on the trochlear groove of the distal femur was created using corneal trephine. Then, suited 3D bioprinted BCS, BMS, or DS scaffolds were implanted matching with the defect. Forced flexion and extension were conducted for the operated knee to confirm the localization of the implanted scaffolds in the defect. Last, the operated knee joint was closed with suture (4-0 thread), and antibiotics were given intramuscularly for prophylactic infection. After the operation, rabbits were allowed to move freely in their single cages and fed with standard food and water. Eight, 12, and 24 weeks later, rabbits were euthanized for further study. The protocol was approved by the local Institutional Animal Care and Use Committee and complied with the *Guide for the Care and Use of Laboratory Animals*, revised in 2010 and published by the National Academy of Sciences.

### Assessment of cartilage repair

Serial sections (4 mm thick) were cut sagittally through the center of the operative site and stained with H&E, toluidine blue, Safranin O and fast green, toluidine blue, alcian blue, and picrosirius red according to standard protocols. Immunohistochemical staining of markers (PRG4, RUNX2, and collagens II and X) for chondrocyte phenotype and microvessel ingrowth (CD31 and α–smooth muscle actin) was conducted according to standard protocols in the generated cartilage tissue sections in different groups compared with the native cartilage. The stained images were taken, and regenerated cartilage thickness (*n* = 6 for each) was calculated for different bioprinted scaffolds using a light microscope. A modified method was used to evaluate the histological repair of articular cartilage defects (*18*).

## SUPPLEMENTARY MATERIALS

Supplementary material for this article is available at http://advances.sciencemag.org/cgi/content/full/6/37/eaay1422/DC1

## Appendix

View/request a protocol for this paper from *Bio-protocol*.
